# Integrated Analysis of Residue Coevolution and Protein Structures Capture Key Protein Sectors in HIV-1 Proteins

**DOI:** 10.1371/journal.pone.0117506

**Published:** 2015-02-11

**Authors:** Yuqi Zhao, Yanjie Wang, Yuedong Gao, Gonghua Li, Jingfei Huang

**Affiliations:** 1 State Key Laboratory of Genetic Resources and Evolution, Kunming Institute of Zoology, Chinese Academy of Sciences, No.32 Jiaochang Donglu Kunming, 650223 Yunnan, China; 2 Department of Integrative Biology and Physiology, University of California, Los Angeles, 610 Charles E. Young Drive East, Los Angeles, California, United States of America; 3 Key Laboratory of Animal Models and Human Disease Mechanisms of Chinese Academy of Sciences and Yunnan Province, Kunming Institute of Zoology, Chinese Academy of Sciences, Kunming, Yunnan 650223, China; 4 Kunming Biological Diversity Regional Center of Instruments, Kunming Institute of Zoology, Chinese Academy of Sciences, Kunming 650223, China; 5 Collaborative Innovation Center for Natural Products and Biological Drugs of Yunnan, Kunming, Yunnan 650223, China; Chinese Academy of Medical Sciences, CHINA

## Abstract

HIV type 1 (HIV-1) is characterized by its rapid genetic evolution, leading to challenges in anti-HIV therapy. However, the sequence variations in HIV-1 proteins are not randomly distributed due to a combination of functional constraints and genetic drift. In this study, we examined patterns of sequence variability for evidence of linked sequence changes (termed as coevolution or covariation) in 15 HIV-1 proteins. It shows that the percentage of charged residues in the coevolving residues is significantly higher than that in all the HIV-1 proteins. Most of the coevolving residues are spatially proximal in the protein structures and tend to form relatively compact and independent units in the tertiary structures, termed as “protein sectors”. These protein sectors are closely associated with anti-HIV drug resistance, T cell epitopes, and antibody binding sites. Finally, we explored candidate peptide inhibitors based on the protein sectors. Our results can establish an association between the coevolving residues and molecular functions of HIV-1 proteins, and then provide us with valuable knowledge of pathology of HIV-1 and therapeutics development.

## Introduction

It has been over 30 years since human immunodeficiency virus (HIV) was first identified as the causative virus of Acquired immune deficiency syndrome (AIDS) [1]. HIV has two types, HIV-1 and HIV-2, which share many features, such as modes of transmission, intracellular replication pathways and clinical consequences [2]. However, HIV-1 is characterized by higher transmissibility and increased likelihood of progression to AIDS [3,4]. Morbidity and mortality rates due to HIV/AIDS are probably the highest in the world, with over 25 million deaths recorded globally while at least 10,000 youths infected every month [5]. Many efforts have been made to prevent or cure HIV infection. In the recent 20 years, diverse antiretroviral drugs were developed in the treatment of HIV infection [6]. Furthermore, devising an effective vaccine to prevent HIV infection or curtail its progression is considered a promising therapeutic approach [7,8]. However, finding an effective, safe HIV vaccine or drug compound is still an ongoing struggle for HIV-1, which is mainly caused by its rapid genetic evolution. In fact, the evolution rate of HIV-1 proceeds is about 1 million times faster than that of the human genome [9], which is well evidenced from the large number of different HIV-1 strains isolated worldwide. Consequently, the high genetic variation leads to the high adaptation of HIV-1 and poses serious challenges for chemotherapy and vaccine development for HIV-1 infection [10,11]. For example, it shows that drug resistance-associated mutations are present in at least 15% to 25% of the HIV population [12]. Besides, mutations within epitopes in HIV-1 have been studied to affect host-virus interaction, with possible implications for immune recognition [13].

Despite the high degree of mutations in the HIV-1 proteins in the setting of antiretroviral therapy, the spectrum of possible virus variants seems to be limited by patterns of amino acid covariation [14]. The amino acid covariation, also known as coevolution, is conceptualized as correlated mutational behavior between columns of a multiple sequence alignment of protein sequences [15]. The structure and function of proteins need to be maintained throughout correlated substitution patterns between intra- and inter-protein residues. Such correlated mutations are suggestive of compensatory changes that occur between entangled residues to maintain protein function. For HIV proteins, the coevolution events should be more important in maintaining their functions or structures or else the high point mutations might result in severe functional inactivity at any time. Understanding what determines the phenotypical impact of these compensatory mutations is important both for planning targeted mutation experiments in the laboratory and for analyzing naturally occurring mutations found in patients. During the recent years, software and method development for assessing amino acid coevolution have made great advances. Using Statistical Coupling Analysis (SCA), Ranganathan et al. detected correlation rules in the WW domain, which describe aspects of the fold architecture going beyond simple protein contacts [16]. Onuchic et al. applied direct coupling analysis (DCA) to genomics-aided structure prediction [17]. With the increase of sequenced HIV protein sequences, we believe the covariation analysis of HIV-1 proteins will be valuable for studying the functions of HIV-1 proteins and anti-HIV therapies.

In this study, we explored all potential coevolution events in HIV-1 proteins. In addition, we applied molecular dynamic simulations to determine the structural features of the coevolving residue pairs. These resides are organized into physically contiguous networks, termed as ‘protein sectors’. We further estimated the association between protein sectors and the functional sites in HIV proteins, such as drug-binding regions, catalytic sites, and epitopes. Our results can establish association between the coevolving residues and molecular functions of HIV-1 proteins.

## Results

### Coevolution events in HIV-1 proteins

**Fig 1:**
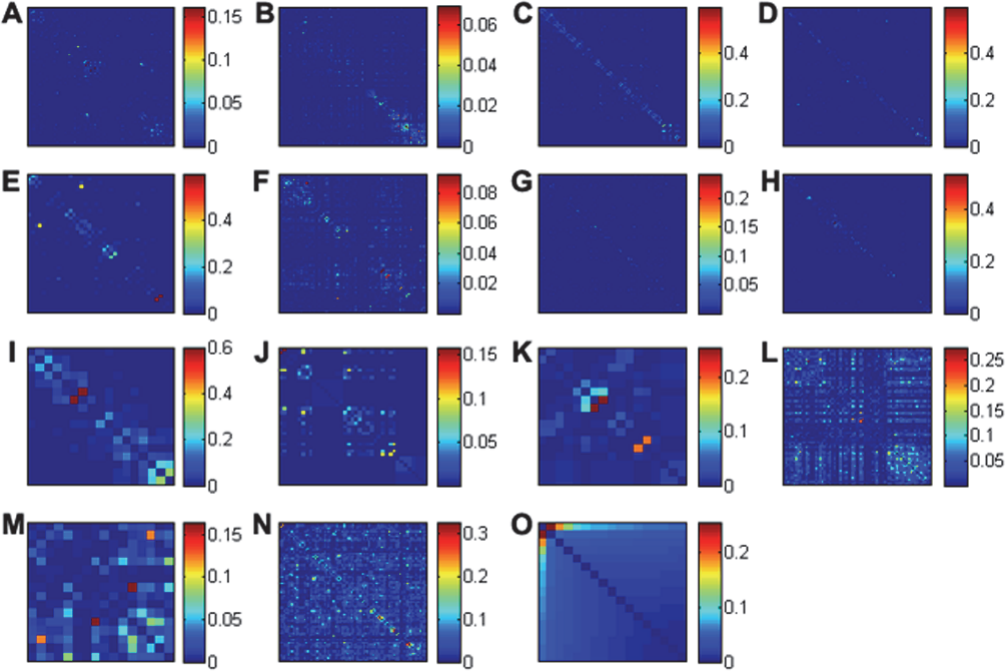
Coevolution patterns in 15 HIV-1 proteins. The panels (A-L) are heat maps of the direct information (DI) values of residue pairs in multiple sequence alignments of GP120, GP41, MA, CA, NC, PR, RT, IN, P6, NEF, REV, TAT, VIF, VPR, and VPU, respectively. The x- and y-axes represent the positions of amino acid residues in the multiple sequence alignments with gap filtering (see Methods).

After multiple sequence alignment and gap filtering, we detected the coevolving residues in the 15 HIV-1 proteins using DCA (Materials and Methods). It shows that the coevolution events exist in HIV-1 proteins (Fig. 1), with count from two (Fig. 1B) to 407 (Fig. 1N). The accessory proteins (P6, NEF, REV, TAT, VIF, VPR, and VPU) have significantly higher mean DI values than the other two groups (Wilcoxon rank sum test, p = 3.11×10^-4^), including viral enzymes and structural proteins. Although the coevolving residues show different patterns among 15 HIV-1 proteins, the majority of them are more proximal in protein sequences compared with random residue pairs (One-sided Two-sample Kolmogorov-Smirnov test followed by 10000 permutations, all p<×10^-2^). In addition, we performed mutual information analysis and SCA on the multiple sequence alignments and found that the coevolving residues detected through DCA tended to show significantly higher Z-scores than the random residue pairs (two-sample student t-test, p = 4.71×10^–5^ and p = 1.10×10^-4^ separately), suggesting that our results were robust to different methods. The percentage of four charged amino acids (including Glu, Asp, Lys, and Arg) in coevolving residues was significantly higher than that in all the proteins (Fisher’s exact test, p = 2.1×10^–3^).

Furthermore, we explored the frequency of the coevolving residue pairs and found that some residue pairs were most abundant, for example Gln-Arg and Arg-Glu. More interestingly, the frequency of the amino acid composition is quite different among different protein categories (Table 1). For the structural proteins, Lys-Glu, and Cys-Cys are the most two abundant pairs, indicating that the presence of salt bridges and disulphide bridges in the HIV-1 structural proteins are critical for their functions.

**Table 1:** The top frequent residue pairs in HIV proteins.

**HIV proteins**	**Structural Proteins**	**Viral Enzymes**	**Accessory Proteins**
Residue pairs	LYS-GLU, CYS-CYS, ASN-THR, LEU-SER, GLU-LEU	ILE-ILE, ASN-LYS, GLY-ALA, ASP-LYS, LYS-GLN	GLN-ARG, ARG-ARG, LEU-ARG, GLN-THR, ARG-GLU

### The protein structural features of coevolving residues

Coevolving residues in several protein families have been proven to work together to enable protein-protein interactions [18], promote folding [16], or contribute to an enzymatic activity [19]. As a result, we explored whether the coevolving residues in HIV-1 proteins showed specific patterns in tertiary structures.

**Fig 2:**
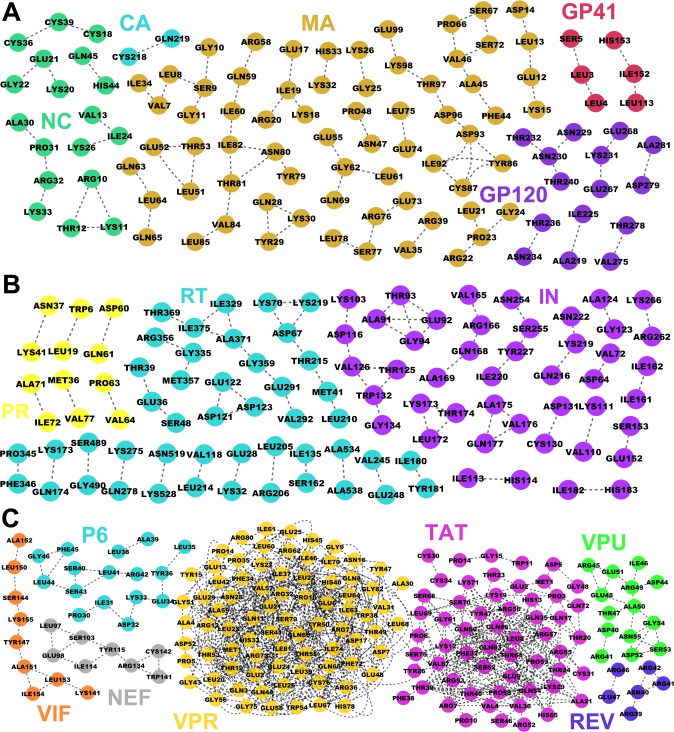
Coevolution networks of HIV-1 proteins. The nodes represent the amino acid residues in HIV-1 proteins while the edges are the coevolving relationships among the residues. The amino acid labels come from the protein tertiary structures (Table 2). The proteins are classified into three categories, including structural proteins (A), viral enzymes (B), and accessory proteins (C).

We mapped the coevolving residues to the selected protein structures (Table 2) and then reconstructed coevolution networks for all the HIV-1 proteins (Fig. 2). Some of the residues in the networks interact with more than one residue, for example, Thr81 in MA (Fig. 2A) and Asp67 in RT (Fig. 2B). Some of the coevolving residues tend to form closely connected modules, for example, residues in VIF (Fig. 2C). In addition, it shows that accessory proteins VPR and TAT cover more resides than other proteins when we set the same criteria for all the proteins. There are two reasons: these two proteins are of short lengths (96 amino acids for VPR while 86~101 for TAT) and they have relatively longer conserved sequences [20].

**Table 2:** HIV-1 protein structures used in the study.

**Categories**	**Proteins**	**Structures**	**Chains**	**Length**	**References**
Structural Proteins	GP120	1G9M	G	321	[25]
	GP41	2CMR	A	226	[59]
	MA	1HIW	A	137	[60]
	CA	3H47	A	231	[61]
	NC	1A1T	A	55	[62]
Viral Enzymes	PR	7HVP	A, B	99	[63]
	RT	1HYS	A	553	[24]
	IN	1EX4	A	212	[64]
Accessory Proteins	P6	2C55	A	52	[65]
	NEF	1AVV	A	130	[66]
	REV	1ETF	B	23	[67]
	TAT	1JFW	A	86	[68]
	VIF	3DCG	E,F	39	[69]
	VPR	1ESX	A	96	[70]
	VPU	1VPU	A	45	[71]

**Fig 3:**
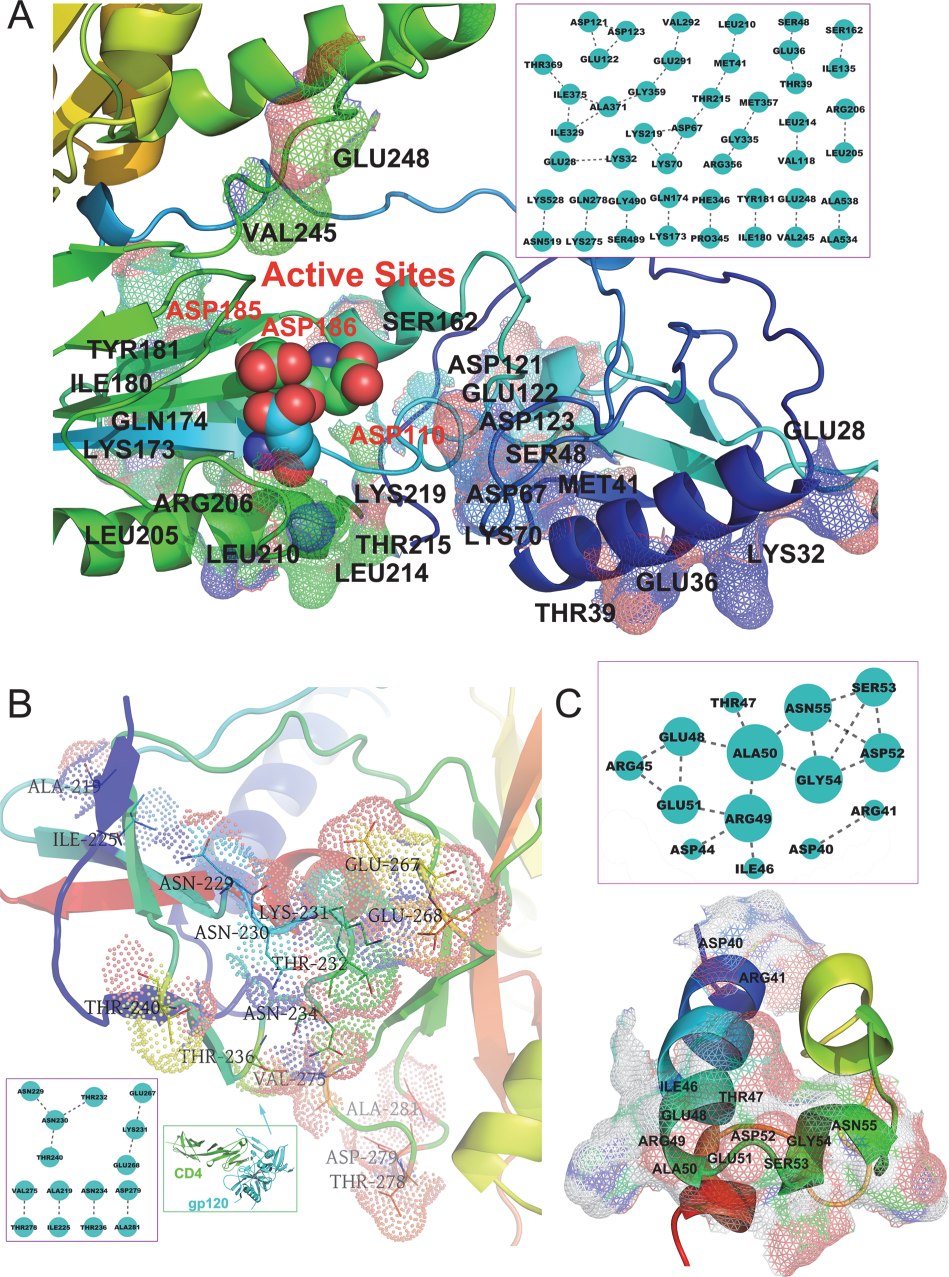
HIV protein sectors underlying conserved, independently varying biological activities. (A) The coevolving residues in RT enzyme were located near the three catalytically essential amino acid residues (Asp110, Asp185, and Asp186) for polymerase catalysis. (B) For gp120, the coevolving residues were located near the protein-protein interface between gp120 and CD40, especially for Glu267, Glu268, Thr278 and Asp279. (C) For VPU protein, 6 out of 12 coevolving residues in the protein sector are charged amino acids. The figures were generated using PyMol (http://www.pymol.Org). The protein structures were colored with a default spectrum of rainbow colors in Pymol. The mesh surfaces of the coevolving residues were added while the different colors correspond to different amino acid residues.

The average distances between the coevolving residues are significantly shorter than that between the random residue pairs (One-sided Two-sample Kolmogorov-Smirnov test followed by 10000 permutations, all p<×10^-6^). It was observed that the coevolving residues in the proteins structures tended to be located proximal to each other, forming relatively independent units. According to previous studies, the similar units in other proteins were termed as “protein sectors” that underlie conserved, independently varying biological activities [21,22]. We found that most of the detected protein sectors in HIV-1 proteins are typically built around protein active sites (Fig. 3, S1 Fig.). For reverse transcriptase (Fig. 3A), there are three opposite charged coevolving residue pairs in the proteins sectors, including Asp67-Lys70, Glu28-Lys32, and Asp67-Lys219, which was located near (within 10 Å) the three catalytically essential amino acid residues (Asp110, Asp185, and Asp186) for polymerase catalysis [23,24]. For gp120 (Fig. 3B), the protein sector was located near the protein-protein interface between gp120 and CD40 [25], especially for Glu267, Glu268, Thr278 and Asp279, suggesting that the protein sector was involved in the HIV entry. For VPU protein, 6 out of 12 coevolving residues in the protein sector are charged amino acids (Fig. 3C). In addition, the mesh surfaces of the coevolving residues suggest that the protein sectors are relatively compact and independent in the HIV-1 protein structures (Fig. 3, S1 Fig.).

### The dynamic behaviors of protein sectors in molecular dynamic simulations

**Fig 4:**
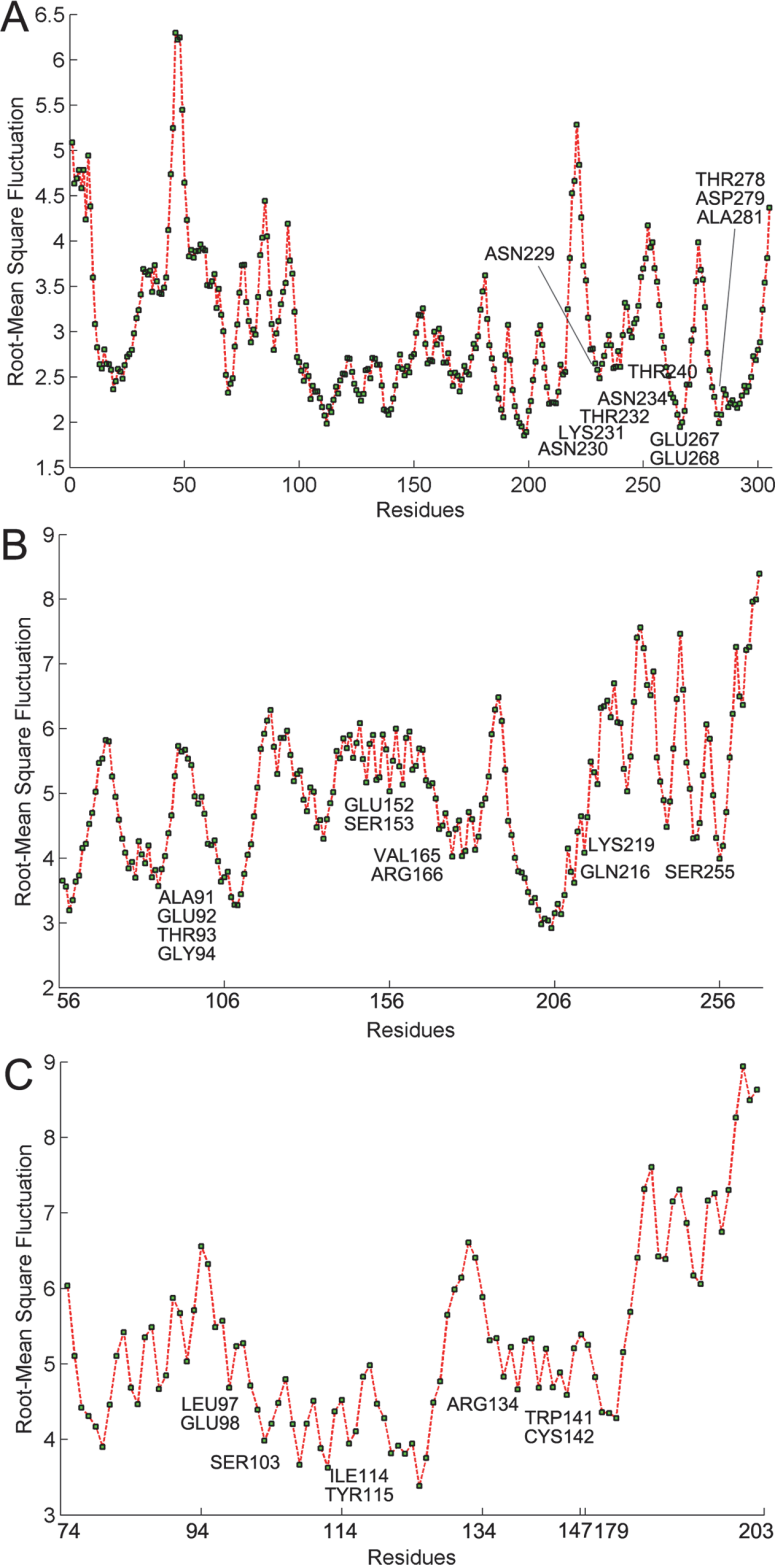
RMSF plot during molecular dynamic simulations. The figure shows backbone RMSF of GP120 (A), IN (B), and NEF (C) in molecular dynamics simulations of 10 ns. The x-axis represents protein sequences while the y-axis is average RMSF values.

**Fig 5:**
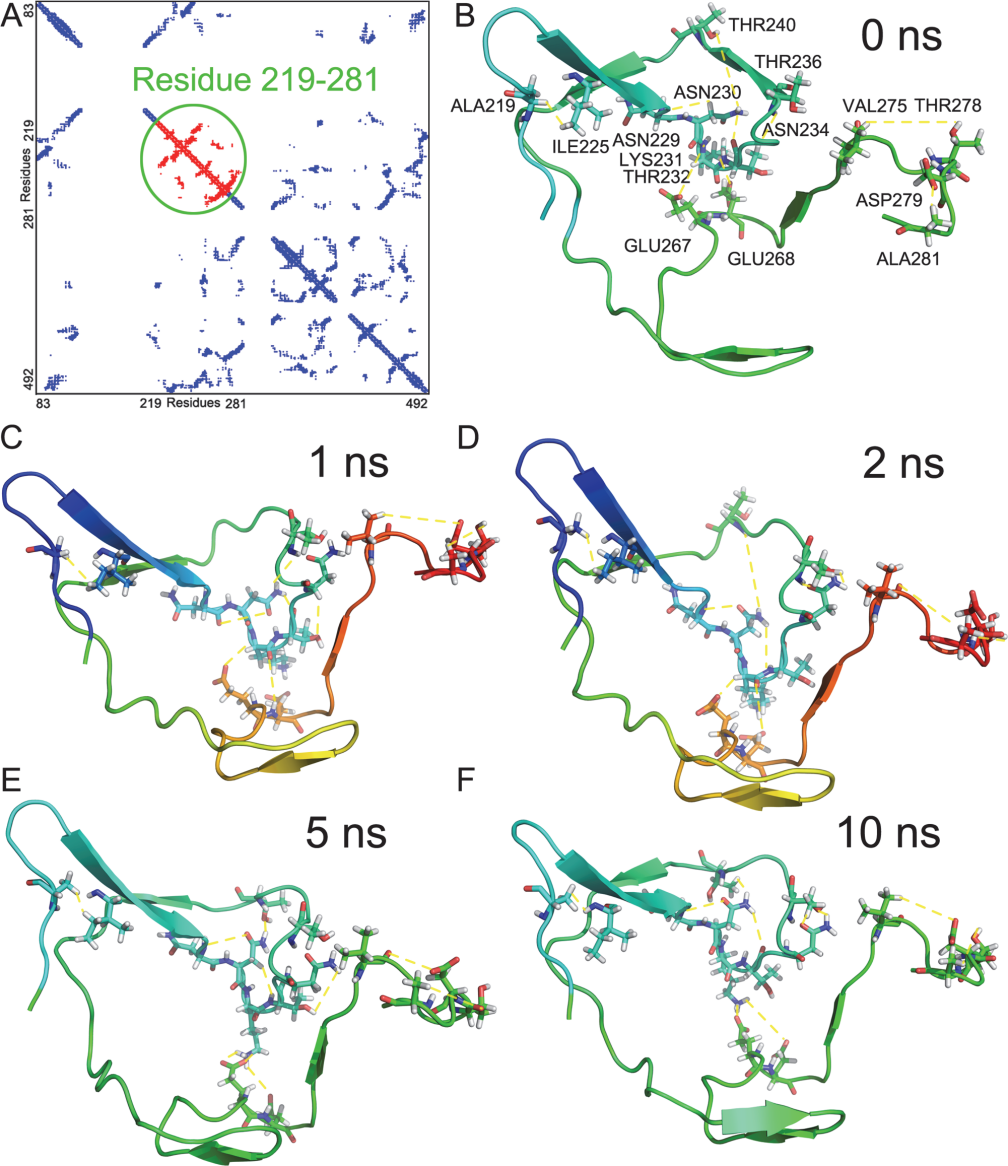
Interactions between coevolving residues in GP120. (A) The contact (hydrogen bonds) maps from molecular dynamics simulation of GP120; (B-F) Snapshots of the interactions between coevolving residues in protein sector of gp120 during molecular dynamics simulations of 0 ns, 1 ns, 2 ns, 5 ns, and 10 ns separately.

Molecular dynamics (MD) simulations are becoming a standard part of workflows in structural biology and enable us study the dynamical properties of a system in full atomic details [26]. Here we applied MD simulations to all the HIV-1 proteins and explored the dynamics behaviors of the proteins sectors in the tertiary structures. The average backbone root-mean square fluctuation (RMSF) for coevolving residues in proteins sectors is significantly smaller than the average RMSF for residues outside protein sectors (Fig. 4A-4C, S2 Fig.; two-sample student’s t-test, p = 7.16×10^-7^), indicating that the protein sectors are significantly stable during the molecular dynamic simulations. However, we observed some exceptions, for example, Leu3, Leu4, and Ser5 in gp41. Recent studies reported that the hydrophobic fusion peptide (FP), where the coevolving residues were located, played important roles in gp41 fusion conformations but did not add stability [27]. In addition, we determined the interactions between coevolving residues during the simulations. To probe the key interactions between coevolving residues in the protein sectors, the contact map was analyzed over the 10 ns molecular dynamics simulations. We take gp120 as an example. It indicates that residues in protein sectors tend to form densely packed substructures (Fig. 5A). Snapshots of molecular dynamics simulation of protein sector in gp120 indicate that the interactions between coevolving residues are stable (Fig. 5B-5F). In addition, it shows that most of the coevolving residues were located in disordered loop structures. For other HIV-1 proteins, the protein sectors also tended to form densely packed substructures in molecular dynamic simulations (S3 Fig.).

### The drug resistance mutations and epitope regions in protein sectors

Drug resistance is a common cause of treatment failure for HIV infection. We explored whether the polymorphisms in the detected protein sectors might be associated with drug resistance. It shows that for almost all the types of HIV antiretroviral drugs, the polymorphisms leading to drug resistance are involved in the coevolving proteins sectors (Table 3). For example, the coevolving residue pairs, ASP67-LYS70 and MET41-THR215 were reported to be the most common mutation patterns for nucleoside RT Inhibitors, including azidothymidine (AZT), Stavudine (d4T), Tenofovir Disoproxil Fumarate (TDF), Abacavir (ABC), Didanosine (DDI), and lamivudine (3TC) [28]. It also shows that the predominant polymorphisms of residue 36 and 77 in protease are the branched chain amino acids (Ile, Val, and Leu), but the transitions among these amino acids resulted in the resistance of protease inhibitors [29]. In addition, it was observed that the many coevolving residue pairs in protein sectors had not been studied in the HIV drug resistance, suggesting that these regions could be served as potential target sites for HIV drugs. For example, two of the six coevolving residues in gp41 have been proven important for the interactions between the C-terminal heptad repeats (CHR) and N-terminal heptad repeat (NHR) domains [30] (S4 Fig.). Moreover, the residues are located in or nearby the peptide HIV Fusion Inhibitors, such as T20 and N36 (S4 Fig.). However, the interactions between coevolving residues are still functionally unknown, which might need site-directed mutagenesis approach to identify the associations between these coevolution events HIV fusion.

**Table 3:** Polymorphisms in protein sectors of HIV-1 proteins leading to drug resistance.

**Coevolving Residues ^a^**	**Top mutation patterns**	**Affected drugs**	**References**
Reverse transcriptase			
41, 67, 70, 210, 215	41L,67N,210W,215Y	Nucleoside reverse transcriptase inhibitors	[46]
	41L,210W,215Y		
	41L		
	67N,70R		
	70R		
	41L,215Y		
	41L,184V		
	67N,70R,215F		
	215Y		
	41L,210W		
	67N		
181	181C	non-nucleoside reverse transcriptase inhibitors	[46]
	181I		
	181V		
Integrase			
92, 153	92Q	Integrase inhibitors	[72]
	153Y		
Protease			
36, 63, 71, 77	36I	Protease Inhibitors	[29]
	36L		
	36V		
	63P		
	71V		
	71T		
	77I		
gp120			
91, 92, 93, 94, 172, 173, 174, 175, 176	Changes within gp120 surrounding the Phe 43 cavity	Entry inhibitors	[73]
	Changes within gp120 (V3, C2, V2 and C4)		
	Changes within gp120 (V3, V1, V2 and V4)		

Note: ^a^ The residue numbering is based on the protein structures in Table 2.

**Fig 6:**
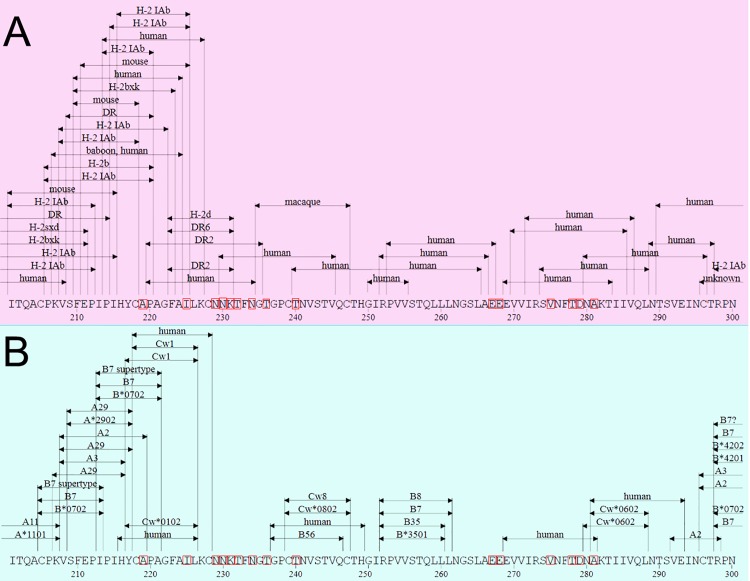
Epitopes of CD4+/CD8+ T lymphocytes for gp120 protein. The epitopes of CD4+ (A) and CD8+ (B) T lymphocytes in protein sector of gp120 protein.

We also investigated the relationships between HIV-1 T cell epitopes and protein sectors. We took gp120 as an example. It shows that protein sectors have overlaps with multiple T cell epitopes (Fig. 6A-6B). We also observed that the sequence from 229 to 236 (NNKTFNGT) was associated with helper T lymphocytes (T-helper/CD4+, Fig. 6A) while not overlapped with cytotoxic T lymphocytes (CTL/CD8+, Fig. 6B). In addition, we searched all the antibodies against gp120 in HIV Molecular Immunology Database (http://www.hiv.lanl.gov/content/immunology). It shows that the protein sectors have more overlaps with antibody epitopes than the other sites (Fisher exact test, p = 3.07×10^–5^).

### The candidate peptide inhibitors of HIV-1 proteins

We detected 33 candidate peptide inhibitors for 12 HIV-1 proteins (Table 4). It shows that most of the peptides (25/33) are of less than 20 amino acids. Most of the interactions between peptide inhibitors and HIV proteins agree with the previous studies [31]. However, the peptide SLLSSPQ (ID: HIP100) was reported to be an integrase inhibitor [32] while was also predicted to interact with gp41. Interestingly, the coevolving residue pair alone can act as effective HIV inhibitors. For example, the peptide DQ (ID: HIP3) was the strongest inhibitor with inhibition constants (Ki) of >1000-fold increase [33]. We can observe that the peptide DQ binds to PR near the protein sector, suggesting that the peptide inhibitor might mimic the ASP60-GLN61 residue pair and then perturbed the functions of the protein sector (S5 Fig.).

**Table 4:** Candidate peptide inhibitors for HIV proteins.

**Potential Targets**	**ID ^a^**	**Sequence**	**Length**	**Mechanisms ^b^**
CA	HIP992	AAAPAATLEEHMTACQGV	18	Maturation
CA	HIP993	ALGAAATLEEMMTACQGV	18	Maturation
GP41	HIP766	RQLLSQIVQQQNNLLRAIEAQQHLLQLT	28	Fusion inhibitor
GP41	HIP763	MTLTVQARQLLSQIVQQQNNLLRAIEAQ	28	Fusion inhibitor
GP41	HIP100	SLLSSPQ	7	Integrase inhibitor
GP120	HIP962	EINCTRPNNNTRKSIRIQRGPGRAFVTIGKIGNMRQAHCNIS	42	Virus entry
GP120	HIP1127	IRKAHCNISRADWND	15	Fusion inhibitor
GP120	HIP1100	GIGDPVTCLKSGAIA	15	Fusion inhibitor
IN	HIP170	ACWWAGIKQEF	11	Integrase inhibitor
IN	HIP171	ACWWAGIRQEF	11	Integrase inhibitor
IN	HIP166	ACWWAGIKQAF	11	Integrase inhibitor
IN	HIP165	ACWWAGIKAEF	11	Integrase inhibitor
MA	HIP625	KRIVQRIKDFLRNLVPRTES	20	Multifunction
MA	HIP185	KRIVQRIKDFLR	12	Multifunction
NC	HIP1148	RSQKEGLHYTCSSHFPYSQYQFWK	24	Fusion inhibitor
NC	HIP1091	RSQKEGLHYTCSSHFPYSQYQFWK	24	Fusion inhibitor
PR	HIP3	DQ	2	Protease inhibition
PR	HIP82	LQITLW	6	Protease inhibition
PR	HIP22	SYEW	4	Protease inhibition
PR	HIP24	SYNL	4	Protease inhibition
PR	HIP20	SFNL	4	Protease inhibition
REV	HIP1168	TRQARRNRRRWRERQRAAAAC	21	Fusion inhibitor
REV	HIP1167	TRQARRNRRRWRERQR	16	Fusion inhibitor
RT	HIP276	ASCDKCQLKGEAMHG	15	Reverse Transcriptase
RT	HIP332	MHGQVDCSPGIWQLD	15	Reverse Transcriptase
RT	HIP616	KELKKIIGQVRDQAEHLKTA	20	Reverse Transcriptase
RT	HIP998	FKLPIQKETWETWWTEYWE	19	Reverse transcriptase
TAT	HIP944	GRKKRRQRRR	10	Virus entry
VIF	HIP360	SVTKLTEDRWNKPQK	15	Blocks multimerization of VIF
VIF	HIP355	RWNKPQKTKGHRGSH	15	Blocks multimerization of VIF
VIF	HIP328	LTEDRWNKPQKTKGH	15	Blocks multimerization of VIF
VPR	HIP253	KGLSGPSEWWVWV	13	VPR
VPR	HIP256	KGLSGPTAWWVVV	13	VPR

Note: ^a^ The peptide IDs are HIPdb ID;

^b^ The Mechanisms were the potential functions of the peptides reported in the previous studies [31].

### More experimental evidence for the functions of HIV-1 protein sectors

Beside the *In silico* evidence described above, recent studies now provide experimental evidence for the functions of the detected sectors in HIV-1 proteins (Table 5). For example, the protein sector in GP120 (residue 275–281in Loop D) was proven to be involved in a loop-based mechanism of CD4-binding-site recognition [34]. The protein sectors in IN enzyme were involved in late-stage event in HIV replication, the disruption of which will lead to the reverse transcription block [35]. Putting all the evidence together, we concluded that the detected protein sectors in HIV-1 proteins are essential during different steps of the HIV life cycle.

**Table 5:** Evidence for molecular functions of protein sectors in HIV-1 proteins.

**Protein Sectors**	**Mechanisms**	**Experimental Approaches**	**References**
GP120	Loop-based mechanism of CD4-binding-site recognition	Co-crystal structure with antibodies	[34]
GP41	Hydrophobic fusion peptide to infect host cell	Monotherapy	[74]
PR	Maintaining structural stability nearby active sites	Crystal structure of multidrug-resistant PR bearing 20 mutations	[75]
IN	Late-stage event in HIV replication	Treatment of virus-producing cell with non-catalytic site integrase inhibitors	[35]

## Discussion

Evolution of HIV-1 proceeds about 1 million times faster than that of the human genome, with approximately one error incorporated into the viral genome each time the virus is replicated [9]. This rapid mutation rate of HIV-1 proteins is widely considered a major stumbling block in the development of therapies to combat acquired immunodeficiency syndrome. To overcome the limitations, we determined the coevolving events in all the HIV-1 proteins and studied their structural features in the study. We found that coevolution showed quite different characteristics among different classes of HIV-1 proteins. The charged amino acids are overrepresented in the coevolving residues. The coevolving residues tend to form protein sectors in tertiary structures, in which interactions between coevolving residues show stable behaviors in the dynamic environment. These protein sectors are closely associated with HIV-1 drug resistance and epitopes. The findings will be helpful in understanding the pathogenesis and developing potential antiviral compounds.

The charged amino acids were enriched in the coevolving residues, suggesting that the interactions mediated by these charged residues are of importance to the functions of HIV-1 proteins. It is universal accepted that the salt bridges in proteins most often arise from the anionic carboxylate (COO-) of negatively charged amino acids (Asp or Glu) and the cationic ammonium/ guanidinium (NH3+ or NHC(NH2)2+) from positively charged amino acids (Lys or Arg) [36]. The salt bridges in HIV proteins mediate the critical activities of the virus, such as entry to host cells [37], replication [38,39], and assembly [40]. Salt bridges are of critical importance for host-virus interactions. Wu et al. found that salt bridges formed between HIV entry inhibitors and CCR5 chemokine receptor, which acts as a co-receptor for HIV-1 viral entry, potentially locked the receptor in an inactive conformation [41]. Therefore, the salt bridges mediated by the charged residues in the coevolving residue pairs will be suitable in designing potential anti-HIV drugs. When drugs disrupt a certain salt bridge in HIV-proteins, there will be four possible endings depending on the type of proteins, including structural collapse, inhibition of host-virus interactions, failure of virus assembly, and loss of catalytic activities. These potential mechanisms of the anti-HIV drugs can be validated by solid experimental evidence, which will be set forth as below. For envelope glycoprotein gp120, two detected coevolving residue pairs (LYS231-GLU267, and LYS231-GLU268) might be involved in the formation of salt bridges. The structural analysis validated that these two interactions were within 5 Å and they together with other coevolving residues formed a protein sector in the inner domain, which was recently proven critical for CD4-required conformational transitions in the HIV-1 Env trimer [42]. For Reverse transcriptase, there are three opposite charged coevolving residue pairs, including Asp67-Lys70, Glu28-Lys32, and Asp67-Lys219, which was located near (within 10 Å) the three catalytically essential amino acid residues (Asp110, Asp185, and Asp186) for polymerase catalysis [23,24]. We presume that the salt bridges formed by these residue pairs play critical roles in maintaining the stability of the catalytic sites. A recent study found that Alizarine derivatives as new dual inhibitors of the HIV-1 reverse transcriptase-associated DNA polymerase and RNase H activities could block the salt bridges by occupying binding pockets near these coevolving residues [43].

More interestingly, the coevolving residues in protein structures tended to form protein sectors, which are closely associated with critical features of HIV proteins, such as active sites, drug resistance, and epitopes. Protein sectors found in other protein families so far were related to conserved functional activities [15], which positively supported our findings. As a result, we will discuss according to the possible functions of the detected protein sectors. The residues in the protein sectors were located near the active sites, representing the structural basis for allosteric communication in proteins [44]. Especially for viral enzymes (IN, PR, and RT), one of the coupled positions is the active site and the other is the allosteric site. It can be easily inferred that binding of HIV inhibitors at the allosteric site will cause conformational changes, which consequently results in modified enzyme activity. In recent drug development studies, targeting allosteric sites of enzymes is becoming increasingly hot topic [45]. The protein sectors are available as potential target sites for allosteric activities. Second, the coevolving residues are observed closely associated with drug resistance. It was reported that over 50 percent of patients under anti-HIV therapies were infected with viruses that show resistance to antiretroviral drugs [46]. The principle mechanisms for drug resistance in anti-HIV treatments are mutations (1) reduce affinity of the inhibitors for the proteins; (2) impair incorporation of nucleoside analogues; or (3) block protein-protein interactions. However, these mutations in protein sectors will not affect the basic functions of HIV proteins. For example, we found that the predominant polymorphisms of residue 36 and 77 in protease are the branched chain amino acids (Ile, Val, and Leu) and the mutations conferred high drug resistance. We presume that these compensatory mutations will have little effects on the interactions between coevolving residues while reduce the affinity between inhibitor and protein or block the access of the drug to protein cavity. At times, a single mutation in the genetic code can confer complete resistance to some antiviral drugs (Table 3), suggesting that the transitions of amino acid do not perturb the functions of proteins sectors. To avoid drug resistance, we can inactivate the whole protein sectors. In addition, we also observed that the sequences in protein sectors were associated with specific epitopes of T lymphocytes (Fig. 6). HIV-1 infection is characterized by CD4+ T cell depletion, CD8+ T cell expansion, and chronic immune activation that leads to immune dysfunction [47]. The HIV-specific CD4+ T cell response can be recovered after initiation of highly active antiretroviral therapy, which is inversely correlated with HIV viral load [48,49]. Therefore, it suggests that the HIV virus might be controlled by a vaccine incorporating improved CD4+ epitopes to induce a stronger CD4+ T cell response for helping HIV-specific CTL proliferation, together with similarly enhanced CTL epitopes [50]. The CD4+ epitopes improved by the integrated approaches of coevolution and structural analysis might be a component of a more effective second generation vaccine construct for HIV infection.

Finally, we also detected several candidate HIV inhibitory peptides. The development of drugs for HIV infection began soon after the virus was discovered 30 years ago, during which peptide inhibitors had shown budding potential to exploit HIV proteins as targets for intervention. Peptide inhibitors possess several advantages over traditional anti-HIV drugs [51]: First, peptides have little toxic side effects for their specificity; second, peptides have more diverse targets (Table 4). The detected peptides mimic the interactions between coevolving residues and then disturb the stable substructures maintained by protein sectors. Furthermore, we observed that the peptides could target the key steps involved in virus attachment, fusion and replication etc, offering potentially attractive vaccine targets during immune response to HIV infection [52].

In sum, the integrated analysis captures several key protein sectors in HIV-1 proteins, providing us with valuable knowledge of pathology of HIV-1 and therapeutics development. Although our analysis covers all the HIV-1 proteins, there is still a lot more information to dig out from these results. The functions of these protein sectors should be further validated in the process of rational vaccine design and development of diagnostical tools. A greater understanding of the functions of HIV protein sectors may be critical in anti-HIV research.

## Methods

### HIV-1 protein sequences and alignment

The HIV genome has nine open reading frames (ORF, leading to nine primary translation products, including ENV, GAG, NEF, POL, REV, TAT, VIF, VPR, and VPU) but 15 proteins are made in all as a result of cleavage of three of the primary products. Among these ORFs, The primary ENV product is the protein GP160, which is cleaved to GP120 and GP41. The GAG protein is synthesized as a polyprotein in the cytosol of an infected cell, and contains four functional segments: MA, CA, NC, and p6. The three POL proteins, PR (protease), RT (reverse transcriptase), and IN (integrase), provide essential enzymatic functions and are encapsulated within the particle. The sequences of 15 HIV-1 proteins were retrieved from HIV Sequence Database (http://www.hiv.lanl.gov/), with each containing over 2000 non-redundant sequences (sequence identity was set to 99% as cutoff). The multiple sequences from the same patient or transmission chains were excluded. We applied MUSCLE program (http://www.drive5.com/muscle/) in the multiple sequence alignment (MSA). After the alignment, the columns with gap ratio >20% were removed. Furthermore, we separated the multiple sequences according to the genetically distinct subtypes in the “main” group M of HIV-1 strains. Based on the perturbations at sites in the MSA [44], we found the homologue sequences in each subtype were inadequate for representing the properties of the HIV proteins.

### Detecting co-evolving residues

The MSA for each HIV-1 protein was analyzed using direct coupling analysis (DCA), is a statistical inference framework used to infer direct co-evolutionary couplings among residue pairs [15]. The main output, direct information (DI) values for all column pairs, is a measure of the direct coevolutionary coupling between residue positions. High DI was previously shown to be an accurate predictor for residue–residue contacts [15]. We determined the coevolving residues as the top residue pairs with DI>0.05. Simultaneously, the mutual information between amino acid residues were calculated using the DCA program.

Besides, the MSA for each HIV-1 protein were analyzed by using the statistical coupling analysis (SCA) method [53]. The SCA correlation matrix between amino acids was turned into Z-scores (also called Standard scores). If a Z-score was above a fixed threshold (cutoff = 4), two corresponding sites were linked by an edge, and each site was represented as a node.

### Selection of protein structures

All the HIV-1 protein structures were obtained from RCSB PDB database (http://www.rcsb.org/pdb/), which stored more than 2000 HIV-1 protein structures (as to Jan 28, 2014). We set the following criteria to select the proper protein structures for the following analysis. First, the selected structure showed the highest sequence coverage. Then, we performed pairwise alignments between the query sequence (from the PDB file and chain ID) and every sequence in an MSA (alignment) to find the top hit sequence. Finally, a residue number list that relates alignment numbering to structure numbering was generated.

### Molecular dynamic simulations

All simulations were performed using NAMD 2.8 [54] and the CHARMM31 force field with CMAP correction [55,56]. The ionized systems were minimized for 50,000 integration steps and constrained equilibrated for 10 ns with 2 fs time stepping and frames stored each picosecond. Constant temperature (T = 310 K) was enforced using Langevin dynamics with a damping time constant of 5 per picosecond. Constant pressure (p = 1 atm) was enforced through the Nosé-Hoover Langevin piston method with a decay period of 100 fs and a damping time constant of 50 fs. Van der Waals interaction cutoff distances were set at 12 Å (smooth switching function beginning at 10 Å) and long-range electrostatic forces were computed using the particle-mesh Ewald (PME) with a grid size of less than 1.0 Å.

### Data sets of HIV-1 drug resistance and epitopes

To examine the enrichment of the functional residues in HIV-1 proteins in the coevolving residues, we collected the information of HIV-1 drug resistance and epitopes for all the HIV-1 proteins. The drug resistance data sets were retrieved from HIV Drug Resistance Database (http://hivdb.stanford.edu/) and the epitopes information was obtained from HIV Molecular Immunology Database (http://www.hiv.lanl.gov/content/immunology). Because HIV Drug Resistance Database contains information for only several HIV proteins (PR and IN), we complemented the information for other proteins through text-mining approach from the PubMed database. In addition, we retrieved base-by-base details of the landmarks of HIV-1 proteome (http://www.hiv.lanl.gov/content/sequence/HIV/MAP/annotation.html).

### Screening peptide library

We first collected the candidate HIV inhibitory peptides from HIPdb database [31]. We built up a structure library for these peptides using Open Babel toolbox (version 2.3.1) [57]. Then, we performed screening peptide library against all the 15 HIV proteins using LibDock [58]. In the molecular docking process, we set the coevolving residues in protein sectors as interaction sites with default parameters in LibDock. Then, the resulting peptides were further evaluated using coevolving residue pairs. For example, the resulting peptides for REV protein should contain patterns like Arg-Asn/ Asn- Arg, Arg-Arg, or Arg-Glu.

### Statistical analysis

Two-sample student’s t-test was performed to compare the coevolving residue pairs and random residue pairs in coevolution calculation and molecular dynamic simulations for each HIV-1 protein. We performed Wilcoxon rank-sum test to compare the coevolution patterns between HIV proteins. All the statistical analysis was done using R.

## Supporting Information

S1 Fig Protein sectors in HIV-1 proteins. The panels (A-L) represent CA, NEF, IN, GP41, PR, REV, MA, TAT, NC, VIF, VPR, and P6 respectively. Only part of the coevolving residue pairs was labeled. The detailed coevolution events were listed in Fig. 2.
(PDF)

S2 Fig Root-mean square fluctuation (RMSF) of residues in HIV proteins during the molecular dynamics (MD) simulations. Panel A-L represent CA, GP41, MA, NC, P6, PR, REV, RT, TAT, VIF, VPR, and VPU respectively. For each protein, X axis is the sequence of the protein structure in Table 2 while Y axis is the average RMSF during the 10 ns molecular dynamic simulations.
(PDF)

S3 Fig Contact map of HIV proteins during molecular dynamic simulations. Panel (A-N) represent CA, GP41, IN, MA, NC, NEF, P6, PR, REV, RT, TAT, VIF, VPR, and VPU respectively. The red regions are the protein sectors. For TAT and VPR, only the top ten coevolving residues were marked.
(PDF)

S4 Fig Schematic representation of the coevolving residues, functional domains, and peptide fusion inhibitors in gp41. The black dashed lines between NHR and CHR indicate interactions between the residues located at the e and g positions in the NHR and at the a and d positions in the CHR. The residues at the a and d sites in the CHR helical wheel are important for formation of the internal trimer by NHR domains while the residues at the e and g sites in the NHR helical wheel are involved in interactions between the NHR and CHR domains that result in the formation of six-helix bundle. The numbers of residues of peptides corresponding to T21, N36, T20, C34, and CP32M are shown. The red dashed lines represent the detected coevolution events in gp41. The pocket-forming sequence in the NHR domain, the pocket-binding domain (PBD), GIV-motif-binding domain (GBD), and lipid-binding domain (LBD) in the CHR domain are highlighted in purple, green, blue, and orange, respectively. The gp41 reference sequence in the figure was retrieved from [30].
(PDF)

S5 Fig The docking results between HIV-1 protease and DQ peptide inhibitor. The stars represent conformation clusters of DQ peptide.
(PDF)
