# Correction: Integrated Analysis of Residue Coevolution and Protein Structures Capture Key Protein Sectors in HIV-1 Proteins

**DOI:** 10.1371/journal.pone.0123493

**Published:** 2015-03-30

**Authors:** The PLOS ONE Staff

There are errors in the Funding section. The correct funding information is as follows:

This work was supported by the grants from National Basic Research Program of China (2013CB835100), National Natural Science Foundation of China (31123005), the Instruments Function Deployment Foundation of CAS (Grant Nos.yg2010044 and yg2011057), and West Light Foundation of The Chinese Academy of Sciences (awarded to Yuqi Zhao; Grant Nos. Y302401081 and Y302401082). The funders had no role in study design, data collection and analysis, decision to publish, or preparation of the manuscript.
