# Supplementary material for: Integrated Analysis of Residue Coevolution and Protein Structures Capture Key Protein Sectors in HIV-1 Proteins
Source: PLoS One. 2015 Feb 11;10(2):e0117506. doi: 10.1371/journal.pone.0117506 (PMC4324911; doi:10.1371/journal.pone.0117506)
Supplement: S1 Fig — The panels (A-L) represent CA, NEF, IN, GP41, PR, REV, MA, TAT, NC, VIF, VPR, and P6 respectively. Only part of the coevolving residue pairs was labeled. The detailed coevolution events were listed in Fig. 2. (PDF) [file pone.0117506.s001.pdf]

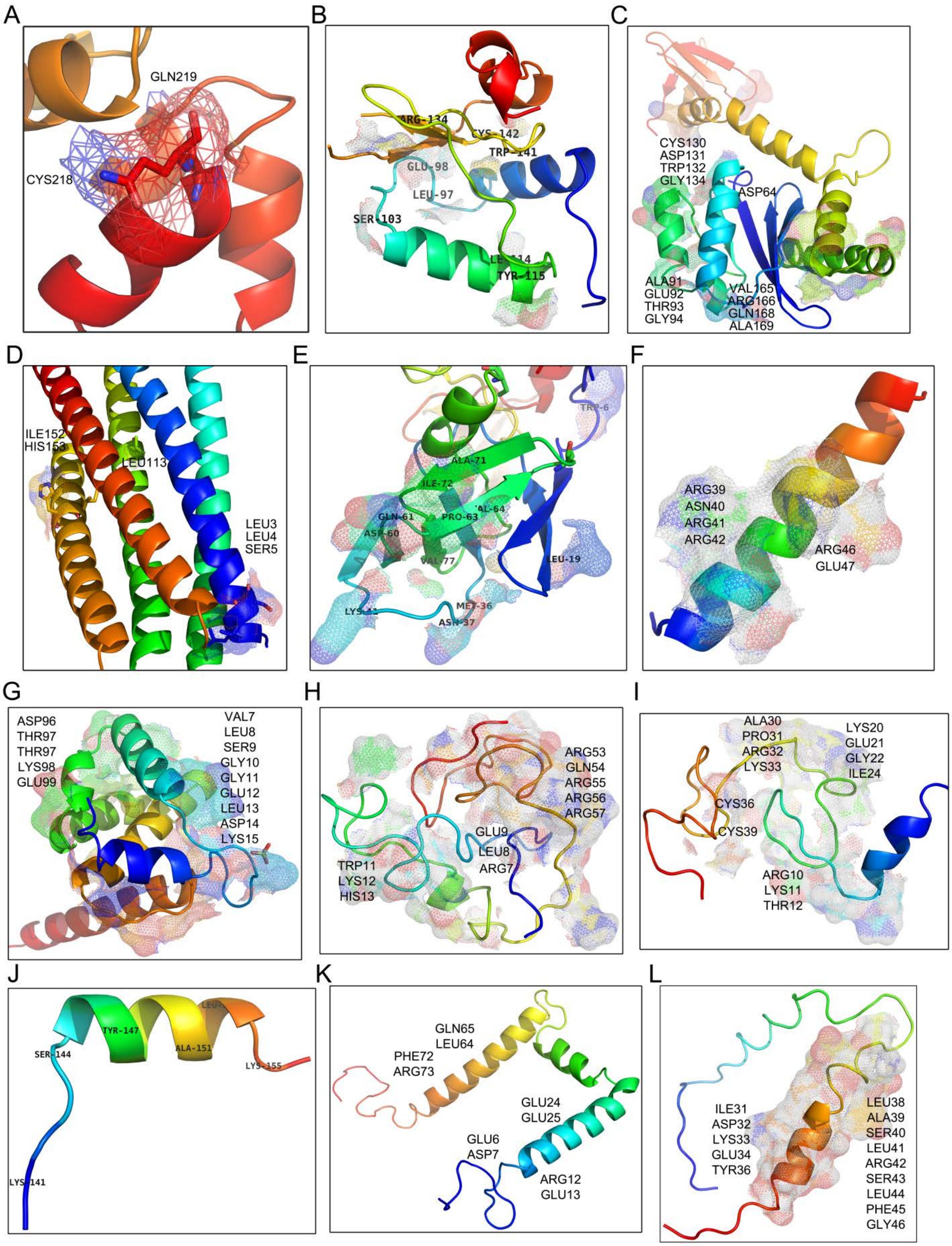
A
GLN219
CYS218
B
ARG-134
CYS-142
TRP-141
GLU-98
LEU-97
SER-103
TYR-115
C
CYS130
ASP131
TRP132
GLY134
ASP64
ALA91
GLU92
THR93
GLY94
VAL165
ARG166
GLN168
ALA169
D
ILE152
HIS153
LEU113
LEU3
LEU4
SER5
E
TRP-6
ALA-71
ILE-72
GLN-61
PRO-63
ASP-60
VAL-77
LEU-19
MET-36
ASN-37
F
ARG39
ASN40
ARG41
ARG42
ARG46
GLU47
G
ASP96
THR97
THR97
LYS98
GLU99
VAL7
LEU8
SER9
GLY10
GLY11
GLU12
LEU13
ASP14
LYS15
H
ARG53
GLN54
ARG55
ARG56
ARG57
GLU9
LEU8
ARG7
TRP11
LYS12
HIS13
I
ALA30
PRO31
ARG32
LYS33
LYS20
GLU21
GLY22
ILE24
CYS36
CYS39
ARG10
LYS11
THR12
J
TYR-147
SER-144
ALA-151
LYS-155
LYS-141
K
GLN65
LEU64
PHE72
ARG73
GLU24
GLU25
GLU6
ASP7
ARG12
GLU13
L
ILE31
ASP32
LYS33
GLU34
TYR36
LEU38
ALA39
SER40
LEU41
ARG42
SER43
LEU44
PHE45
GLY46

Supplementary Figure S1. Protein sectors in HIV-1 proteins. The panels (A-L) represent CA, NEF, IN, GP41, PR, REV, MA, TAT, NC, VIF, VPR, and P6 respectively. Only part of the coevolving residue pairs was labeled. The detailed coevolution events were listed in Figure 2.
