# Supplementary material for: Integrated Analysis of Residue Coevolution and Protein Structures Capture Key Protein Sectors in HIV-1 Proteins
Source: PLoS One. 2015 Feb 11;10(2):e0117506. doi: 10.1371/journal.pone.0117506 (PMC4324911; doi:10.1371/journal.pone.0117506)
Supplement: S2 Fig — Panel A-L represent CA, GP41, MA, NC, P6, PR, REV, RT, TAT, VIF, VPR, and VPU respectively. For each protein, X axis is the sequence of the protein structure in Table 2 while Y axis is the average RMSF during the 10 ns molecular dynamic simulations. (PDF) [file pone.0117506.s002.pdf]

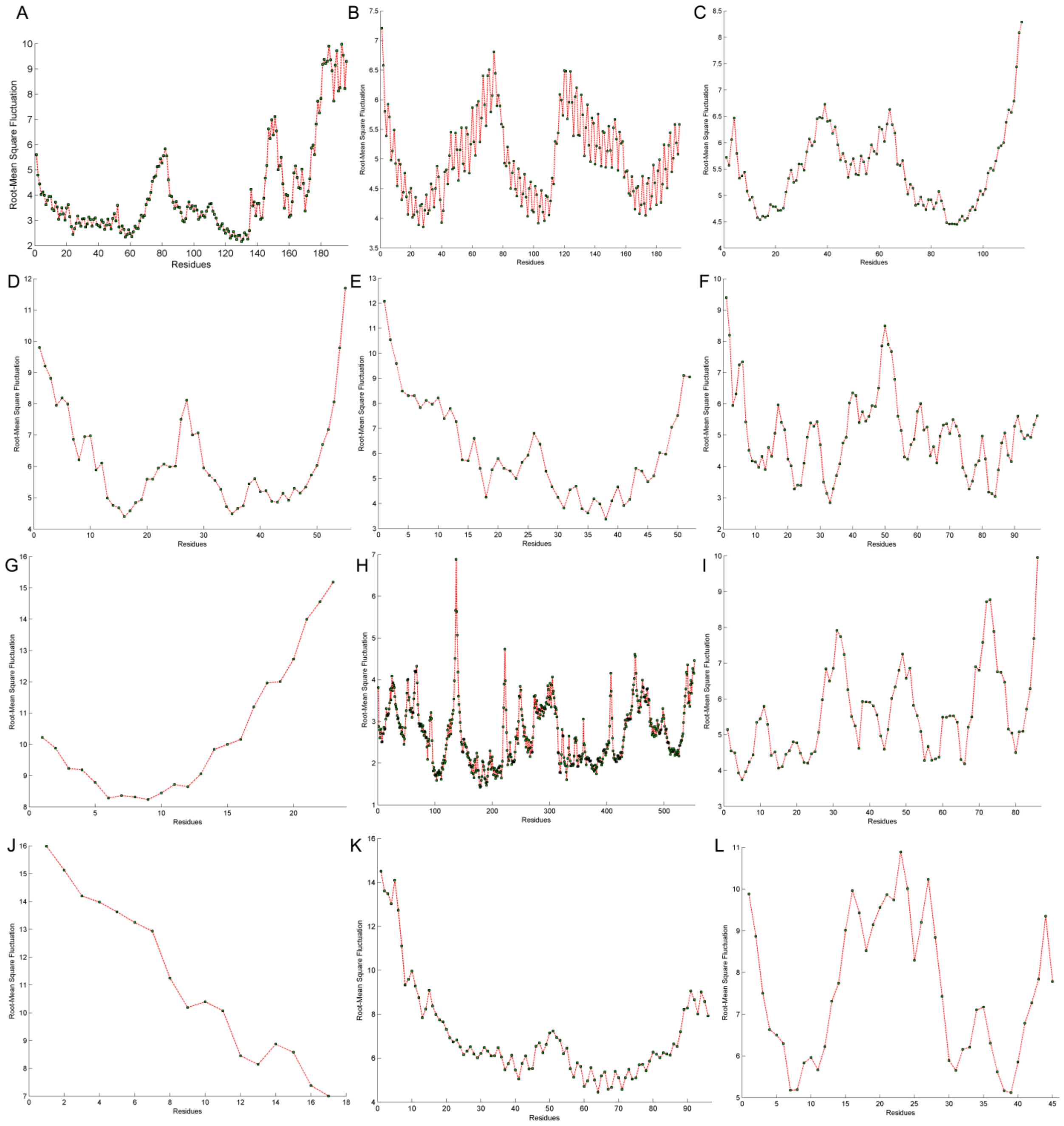

Supplement Figure S2. Root-mean square fluctuation (RMSF) of residues in HIV proteins during the molecular dynamics (MD) simulations. Panel A-L represent CA, GP41, MA, NC, P6, PR, REV, RT, TAT, VIF, VPR, and VPU respectively. For each protein, X axis is the sequence of the protein structure in Table 2 while Y axis is the average RMSF during the 10 ns molecular dynamic simulations.
