# Supplementary material for: Integrated Analysis of Residue Coevolution and Protein Structures Capture Key Protein Sectors in HIV-1 Proteins
Source: PLoS One. 2015 Feb 11;10(2):e0117506. doi: 10.1371/journal.pone.0117506 (PMC4324911; doi:10.1371/journal.pone.0117506)
Supplement: S3 Fig — Panel (A-N) represent CA, GP41, IN, MA, NC, NEF, P6, PR, REV, RT, TAT, VIF, VPR, and VPU respectively. The red regions are the protein sectors. For TAT and VPR, only the top ten coevolving residues were marked. (PDF) [file pone.0117506.s003.pdf]

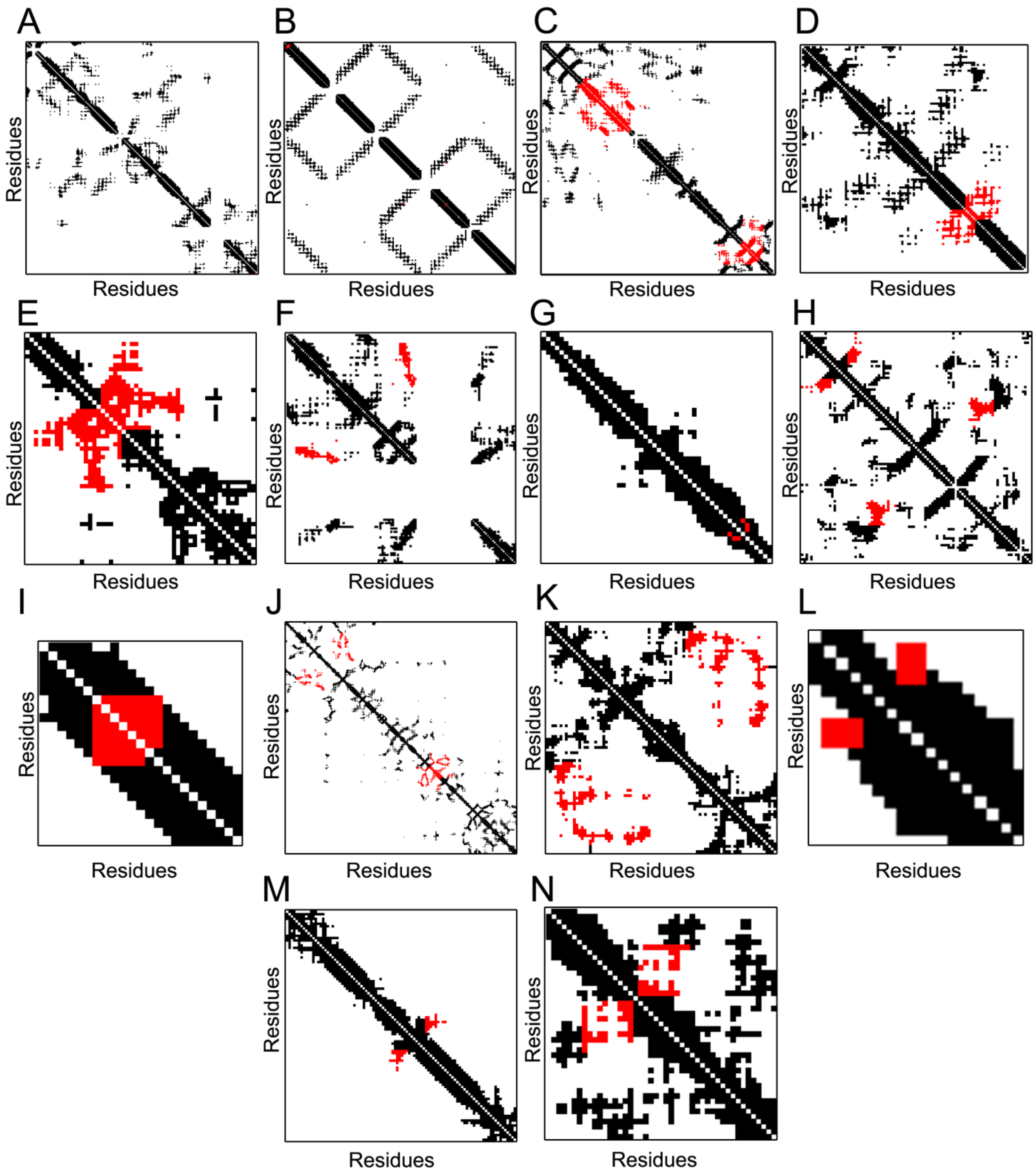

Supplement Figure S3. Contact map of HIV proteins during molecular dynamic simulations. Panel (A-N) represent CA, GP41, IN, MA, NC, NEF, P6, PR, REV, RT, TAT, VIF, VPR, and VPU respectively. The red regions are the protein sectors. For TAT and VPR, only the top ten coevolving residues were marked.
