# Supplementary material for: Integrated Analysis of Residue Coevolution and Protein Structures Capture Key Protein Sectors in HIV-1 Proteins
Source: PLoS One. 2015 Feb 11;10(2):e0117506. doi: 10.1371/journal.pone.0117506 (PMC4324911; doi:10.1371/journal.pone.0117506)
Supplement: S4 Fig — The black dashed lines between NHR and CHR indicate interactions between the residues located at the e and g positions in the NHR and at the a and d positions in the CHR. The residues at the a and d sites in the CHR helical wheel are important for formation of the internal trimer by NHR domains while the residues at the e and g sites in the NHR helical wheel are involved in interactions between the NHR and CHR domains that result in the formation of six-helix bundle. The numbers of residues of peptides corresponding to T21, N36, T20, C34, and CP32M are shown. The red dashed lines represent the detected coevolution events in gp41. The pocket-forming sequence in the NHR domain, the pocket-binding domain (PBD), GIV-motif-binding domain (GBD), and lipid-binding domain (LBD) in the CHR domain are highlighted in purple, green, blue, and orange, respectively. The gp41 reference sequence in the figure was retrieved from [30]. (PDF) [file pone.0117506.s004.pdf]

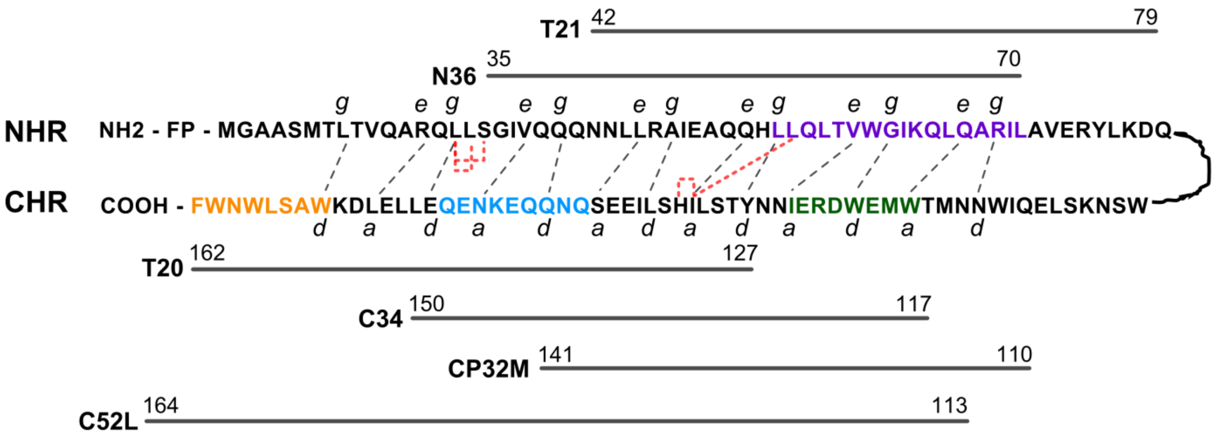

Supplement Figure S4. Schematic representation of the coevolving residues, functional domains, and peptide fusion inhibitors in gp41. The black dashed lines between NHR and CHR indicate interactions between the residues located at the e and g positions in the NHR and at the a and d positions in the CHR. The residues at the a and d sites in the CHR helical wheel are important for formation of the internal trimer by NHR domains while the residues at the e and g sites in the NHR helical wheel are involved in interactions between the NHR and CHR domains that result in the formation of six-helix bundle. The numbers of residues of peptides corresponding to T21, N36, T20, C34, and CP32M are shown. The red dashed lines represent the detected coevolution events in gp41. The pocket-forming sequence in the NHR domain, the pocket-binding domain (PBD), GIV-motif-binding domain (GBD), and lipid-binding domain (LBD) in the CHR domain are highlighted in purple, green, blue, and orange, respectively. The gp41 reference sequence in the figure was retrieved from [30].
