# Supplementary material for: Integrated Analysis of Residue Coevolution and Protein Structures Capture Key Protein Sectors in HIV-1 Proteins
Source: PLoS One. 2015 Feb 11;10(2):e0117506. doi: 10.1371/journal.pone.0117506 (PMC4324911; doi:10.1371/journal.pone.0117506)
Supplement: S5 Fig — The stars represent conformation clusters of DQ peptide. (PDF) [file pone.0117506.s005.pdf]

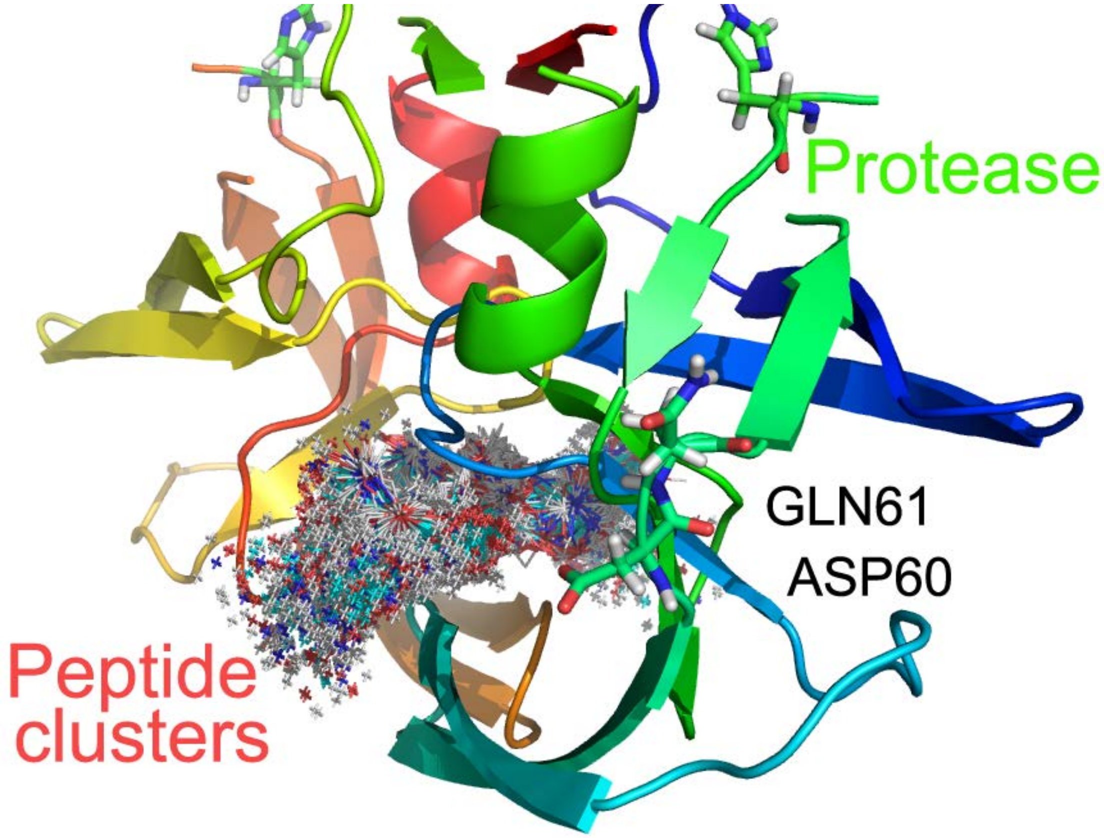

Supplement Figure S5. The docking results between HIV-1 protease and DQ peptide inhibitor. The stars represent conformation clusters of DQ peptide.
